# Reviews

**Published:** 1886-10

**Authors:** 


### REVIEWS.

ZUR MEDICINISCHEN STATISTIK (A contribution to medical statistics), by G. Apella, Doctor and member of several learned societies. Berlin, 1886, Published by H. S. Hermann.

This is an at once singular and interesting pamphlet, somewhat unsystematically written, but withal containing in a short space of thirty-six pages, a large number of condensed facts not generally accessible to the medical or veterinary reader. Beginning with the cardinal statement that statistics will be the foundation of the more exact medical science of the future, the author reviews all the conflicting evidences and theories that have interfered with a proper appreciation of what really is hydrophobia. One draw back about the work is that if the author has arrived at any conclusions, he has succeeded in clothing them in such ambiguous language that they are destined to remain as great a mystery to his readers as they may have been before they picked up the pamphlet. Another peculiar feature is that it is written in four languages; it opens in German, runs on for several pages in English, and winds up with an unhappy *melange* of French, Latin and German. Indeed the last page concludes with a sort of apostrophe as follows, in capitals:

DIE TOLLEN HUNDE
oder
(in des Berichtstatters Version)
DIE BANDE DER THORICHTEN HUNDE
als "Meniss, Ochka, Ochata"
(les chiens fols).

Many will doubtless draw very positive conclusions from this singular linguistic and typographical performance. But aside from his extravagances, the author has laid us under a real obligation. He has applied the foot-rule of exact evidence, to the claims of various investigators, and found the vast majority of them wanting in important qualifications essential to such work as they undertook. On the fourth page he reproduces the "Chronological Table of Outbreaks of Rabies," from the report of the Special Commission appointed by the Medical Press and Circular. To these he adds the following valuable figures of 233 persons bitten in Zurich during 42 years by alleged mad-dogs, only 4 died. Of 106 bitten during the Stockholm epidemic of 1842, only 1 required medical treatment.* In connection with these facts he refers to the suggestive claim that spontaneous hydrophobia originating from the bites of persons while infuriated *or in love*, are to be accounted for as due to trismus and tetanus. We are informed that nearly half a century ago, clear-headed logicians and surgeons, criticized and condemned the specific theory of hydrophobia. The distinguished botanist, Schleiden, inclined to regard Schomann's proposal to cure hydrophobia by extirpating a little vesicle at the base of the tongue† as a form of madness itself.

Dr. Apella says very justly: "The sufficiently uncertain diagnosis of rabies in the dog becomes altogether precarious in the cases of madness of wolves, foxes, cats, sheep, (*Moller*), horses (*Greve*), asses (*Coleius Aurelianus*), camels (*Finke*), etc., increasing in the case of the lyssa of those wounded by infuriated animals (*Galen*) * * * * * * so that it often seems madly confounded whether the animal that bit, were really mad, or the man became mad, because treated for the bite of an alleged mad dog. The remarkable apparatus of an Austrian Count,‡ devised for sufferers from lyssa, is thus described: "A cage with iron bars, a mask for the face with glasses over the eyes, muffs, soaked in oil or wax, sleeves and mantle of oil-cloth, etc.

Among the singular symptoms referred to hydrophobia, the following may interest our readers: Barking like dogs, mewing like cats, tendency to bite after being newly married, *small dogs developing in the urine.* The absurdities around the question of lyssa, recorded by recent opponents of its specific character, are supplemented by the record of an epidemic of rabies among the foxes near Frankfort in 1780, and which was traced to a pig which became *spontaneously rabid.* This is the first intimation we have received that a hog could rival hounds in a fox-hunt.

In recapitulating the various specifics against hydrophobia, the author fairly rants in justifiable sarcasm. A secret nostrum purchased for $200 from the family of Schmie de Kamp, in whose possession it had been for

---

*Of 4,000 cases of bite, treated in the long history of the St. George's Hospital, "it was not known that any of these subsequently developed the disease." This experience is stated in parallelism with Youatt's observation, who failed to find a single case among 400 persons bitten by "mad dogs." They probably had implicit faith in cauterization.

†And yet this form of insanity broke forth again in the person of Marochetti, who claimed to cure lyssa by cutting out not one but several such vesicles, subsequently proven to have no existence.

‡Graf Salm-Reiffenscheid.

200 years, was composed of barley gruel.* We also learn that Dr. Lawrence had much better results than Pasteur with Sentellaria imported from New Jersey, only three deaths occurring in 850 bitten persons. Our view that typhoid fever often manifests itself under the mask of rabies in the dog, we find we have been anticipated by Kochlin, who expressed this opinion years ago. In a former number of this journal an epidemic of rabies is mentioned as occurring in Iceland, the intestines being found ulcerated and filled with a heavy material. From this "epidemic," we draw the same conclusion that Kochlin drew from his.

An interesting instance from among the numerous ones cited, of the effect of the mind on the body, is the case of the steward of Doctor Fracastoro, who died with terrible suffering before the Doctor's eyes, *because he erroneously believed he had been bitten by a tarantula.*

The predecessors of those who were attempting to quiet popular agitation in the last century, were certainly not mealy-mouthed in denouncing what they considered a humbug. Jesse Foote wrote in 1788 of the attempt of Fabroni to cure lyssa by allowing two poisonous snakes to bite the patient. "I will give my opinion of this procedure in a very few words. I regard it as the highest variety of idiocy, as a shameless insult to common sense, and a prostitution of the journal in which it was published." Dr. Apella glides gently from this subject to what he happily designates as the *coarser* forms of the same *superstition* manifested in the snake-cure. The remedies for lyssa enumerated under this head are the following "powdered ants, badger-soup, calves faeces, brain and comb of a cock, cockoo soup, carrion, blood and cleansed faeces, liver, urine, the worm from the lower vesicle of the tongue of a mad dog, flies, the liver of a male goat, horse-dung, menstrual blood (human), the tail of a shrew, *male* (!!!), oyster-shells, the flesh of the unicorn.

As regards the management of the fully developed disease in man, some horrible records are resurrected by Apella. Thus the *Irish Times*, of May, 18, 1861, contains the account of a beautiful young woman, who was smothered to death between two pillows for rabies, in Tipperary, and the *Guardian* of April, 3, 1867, reports the daughter of one A. Woodruff, of Greenfield, Michigan, as having been disposed of in the same manner.†

Pasteur's principle, if not his exact method of curing the disease, with a "bit of hair of the dog that bit you," was anticipated by the Doctors of Finnland, who recommended the blood of the rabid animal to be drunk as a specific against the disease. This treatment can be traced back to Scandinavian sagas, just as the symptoms of traditional lyssa are referable to the "Wahrwolf's Glaube," of the middle ages.

---

*These nostrums are efficacious just as long as they are kept a secret. They lose their virtue when published. The same is true of Radway's Ready Relief and Ayer's pills to this very day. In 1777 a government, not mentioned by Apella, purchased for 10,000 thalers a secret remedy of a peasant, which consisted of a powdered beetle.

†If any reader of the Journal, can furnish confirmatory or contradictory evidence regarding these or similar cases, they will confer a favor on us.

We regret that our researches anent the effect of non rabic inoculation, otherwise following Pasteur's method, were not matured before Dr. Apella's pamphlet was written. We have been unfortunate enough to produce a virus, which increasing in virulence with each inoculation, will, inserted in the brain cortex, or under the dura ot the healthiest and strongest dog, produce death in coma, within twelve hours. It was obtained in the following way.

Among numerous inoculations one had been made with cancerous material from a horse's foot, cleaned and comminuted in sterilized *bouillon.* The dog died the following day in convulsions, which lasted over twelve hours. During these a large amount of saliva escaped from the animal. The floor around it was wetted with it, and the animal was literally bathed in its own discharges. Some of it was caught from the mouth several hours before death. It was tenacious and could not be filtered. This was injected in the brain of a little dog. The animal recovered rapidly from the operation, and seemed normal. But a few hours later it sank, gradually, dying during the night, a peculiar howl being noted. The brain showed no coarse lesion, but its substance had a peculiar bluish-purplish tinge near the site of the injection. The needle had just touched the cortex. The part thus discolored was comminuted, and two drops of the juice injected in the brain of a powerful coach-dog. The operation was rapidly and neatly completed. The next morning this dog was also found dead. The brain appeared disintegrated most remarkably on both sides, but mostly on the side of the injection it was mottled, purplish and blue. The ventricular lining of the lateral ventricles to the depth of two millimetres was exactly in the same condition. A large area of white brain matter, immediately underneath the injection, was converted into a chocolate-colored mass, down to the ventricle. From this brain a small part of the firm substance was treated in like manner, and injected into a third powerful dog, two hours after the former dog's death. This animal recovered in like manner from the operation, and began to sink six hours later, developing a peculiar howl, and dying about midnight, or in nine hours. A fourth dog injected with material from the second dog kept in test tubes, according to Pasteur's method for twenty-four hours, proved nearly as rapidly fatal. All the dogs showed the same symptoms; all died within twenty-four hours, and all showed the same rapid disintegration of brain substance. We have always held that the method of inoculating the brain was a faulty one, for it introduces too many complicating factors; but the rapidity with which the contamination of the brain structures progressed in our last series of cases, indicated some virulent ferment. It remains to be seen whether the hyperdermic injection be followed by the same results, a contingency we are engaged in testing as these lines go to press. S.

A MANUAL OF THE DISEASES OF THE ELEPHANT AND OF HIS MANAGEMENT AND USES. By John Henry Steel, V. S. &c., co-editor of the *Quarterly Journal of Veterinary Science in India*, Madras, 1885.

This unpretentious work comes to us from the very tropics, reminding one of several most plain unattractive looking fruits we remember, which yield in their interior a luscious morsel quite unexpected to the stranger.

Ostensibly this is a veterinary bulletin, for the use of the veterinary surgeons of the British East India Army, but as we open its pages, there appears a table of contents whose items of make up disclose an extended field of treatment, covering the national history of the Elephant; his uses at the present day in the army, though he is admitted to be "getting a little behind times" in warfare; Regulations, Instructions for his welfare in the army, as an animal of transport, Diseases of the Elephant, Medical treatment, &c., &c.

Pages IX to XXV, are devoted to an introduction consisting of a treatise on the Natural History of the Elephant, but including many items of information touching the stock owned by the Government in India. It seems that the Elephant in India, from having undergone domestication during many years has become greatly varied as a species.

Sanderson asserts, and he is regarded as the most notable authority on the subject of this great animal, that there is no evidence of decrease in numbers in India. In some places, as in Ceylon, they are not so numerous as formerly.

A number of varieties are enumerated, each valued or otherwise for certain traits or characteristic features.

An interesting summary of Temminck's conclusion concerning the Sumatran variety, by Prince Lucien Bonaparte indicates this "species" as "perfectly intermediate" between the Indian and African, especially in the shape of the skull, and will certainly put an end to the distinction between *elephas* and *loxodon* with those who admit that antomical genus—"Since" he continues, "although the crowns of the teeth of the *E. Sumatratus* are more like the Asiatic animal, still the less numerous undulated ribbons of enamel are nearly or quite as wide as those forming the lozenges of the African."

The variation in the number of ribs, and the number of the dorsal vertebrae, the sacral, and caudal vertebrae, are interesting facts, and, of course, more radical as differences than those resulting from habits of long continued domestication, whose features form the characteristics so well-known in the various presidencies and sections of India.

Saunderson enumerates a grand total of elephants belonging to the British government in India, at 1,607, besides a few males, kept for state purposes.

Some breeds of elephants have become, during the long series of years they have been under domestication, most happily fitted for certain duties, and for peculiar localities of plain or mountain. In Nepaul the breed of dwarf elephants seem to be especially desirable as adapted to mountain life and travel. Again, in the Malay peninsula there are two varieties, one north of the hilly districts of Queda are very light built, while those south of that region are much more heavily formed.

It is interesting to follow the author's numerous statements of differences. In the latter instance, the elephant is not only more heavy in build, but is more majestic, carries his head higher, and slopes more from the top of the spine to the tail root. The forehead, too, is much more prominent. Another fact is singular, and would seem to be difficult to account for. It is observed that "tuskers" are rare in Ceylon, while in India and south of Queda, in Malaya, they are the rule. Again, some are born with but one tusk. If this be the right hand one, the creature is reverenced by the Hindoos.

Races differ much in tractibility. Those of Northern India, are said to be timid, while those of Southern India are plucky and savage in attack. The females are used by government, as being more reliable, and less subject to excitement and stampede. The males are preferred by the native princes as being more imposing and majestic.

There are five plates, showing figures in outline, very artistically copied from photographs. These are especially valuable for this fact-of being directly from life. The most minute features that are visible at all, are copied correctly. The frontispiece, "Burmese Elephant Moving Beams," would naturally be looked upon as an exaggeration. The enormously large and long beam the creature is bearing off in his trunk's embrace, looks altogether out of proportion, yet the camera is true to its work; and the noble creature must be credited with wonderful power, and intelligence of the first order.

PLATE II. Exhibits two figures, one the baby suckling, and the other the same figures at rest. These have all the excellent effect of good line drawing, with the absolute accuracy of photograph.

PLATE III. Represents two figures of elephants. One is carrying the "Old Fashion Bengal Baggage Gear." The second figure shows a "Shaft Elephant of Heavy Field Battery Gun"—taken from the Artillery Manual. This shows the elephant harnessed much in the same manner as the artillery-horse, with the addition of shafts.

Mr. Sanderson's great assistanee is shown in nearly all the elephant service. There are details here of many improvements introduced by him, in the methods of harnessing.

PLATE IV. Shows two figures: The first being an elephant loaded with tents and the accompanying gear; the great size of the creature, of course, enables the stowage of an entire set of such material.

The second figure of plate IV exhibits the "Gun Elephant." A 9-pounder gun is carried on one elephant, with its carriage and appointments.

Part 3d of the introduction treats of the elephant as a creature of transport. The normal load for the full-sized elephant is calculated as at 800 pounds. Compared with that of camels, the latter carry 240 pounds. That of the 'pack' mule, or bullock, is 200 pounds,

The relative mobility of the elephant as a means of transport is placed high: the beasts moving on forced marches with ease, and at a rate of 3 to 3½ miles an hour, which they keep up for 10 or 12 hours at a stretch. They do with little sleep, and their limbs are especially adapted for long, slow strides, and for rest in the standing position.

Havelock, in marching to the relief of Cawnpore, depended on elephants for conveying 150 of the 5th Fusileers "*in haste*," to the place of mutiny. It is the continued, well sustained marching power of the elephant that proves so valuable in army marching and transportation.

The immense bulk of fodder required on a march is one serious drawback. The elephant requires large range if he must depend on himself for forage.

The author regards the elephant's services as an armor animal at the present day as of far less value than formerly. A serious fault is his liability to illness. He requires great care in many ways.

Part IV treats of the "Pathology of the Elephant," wherein is much interesting reading; though the elephant is not at liberty to become an element in any of our American industries, nor in army economy, this chapter or part is of less interest. The anatomy and physiology of the elephant is well treated, accompanied by some valuable cuts, and diagrams. Full tables relating to Therapeutics are introduced, as, indeed, seemingly every possible needful information relating to the welfare of the great beast. A considerable number of formulas are noticed.

An appendix presents a bibliography of the subject, and a suitable index completes this most valuable and truly interesting volume. Thoroughly valuable to the Eastern owners of elephants, and exceedingly interesting to the general reader. J. B. H.

VETERINARY SURGICAL PATHOLOGY AND PRACTICAL MEDICINE. BY JOHN BURKE AND RICHARD W. BURKE, CAWNPORE, 1886.

Our brethern in the "Far East," have always maintained a position in the front ranks of progressive workers in Veterinary Science. But the authors of the above work, have taken a step in advance, by giving the Oriental native members of the profession, the results of their labors printed in their own tongue. The illustrations are numerous and valuable, many of them being from original designs of the authors.

THE QUARTERLY JOURNAL OF VETERINARY SCIENCE IN INDIA.

We have much pleasure in calling the attention of our readers to this excellent quarterly, edited by veterinary surgeons J. H. Steel and Fred Smith, of the Army Veterinary Department. Sixteen parts have now been issued, and though it is published in India, and is chiefly devoted to animal management and veterinary practice, yet it contains much that is of great value to civil practitioners in this country. The immense territory governed by England in India affords a grand field for investigation and observation—a field which is now only beginning to be cultivated, and through no better channel, led by such able hands, could the accumulating information, coming in from every side, be concentrated, than in this periodical, which contains clinical, botanical, hygienic, and other well written papers, as well as general scientific summaries.--*Veterinary Journal.*

